# Assessments of prolonged effects of desflurane and sevoflurane on motor learning deficits in aged App^NL-G-F/NL-G-F^ mice

**DOI:** 10.1186/s13041-022-00910-1

**Published:** 2022-04-07

**Authors:** Ryo Niikura, Tomoyuki Miyazaki, Kenkichi Takase, Hiroki Sasaguri, Takashi Saito, Takaomi C. Saido, Takahisa Goto

**Affiliations:** 1 Department of Anesthesiology, Yokohama City University Graduate School of Medicine, Yokohama, Japan; 2 Department of Physiology, Yokohama City University Graduate School of Medicine, Yokohama, Japan; 3 Laboratory of Psychology, Jichi Medical University School of Medicine, Simotsuke, Tochigi Japan; 4 Laboratory for Proteolytic Neuroscience, RIKEN Center for Brain Science, Wako, Saitama Japan; 5 Department of Neurocognitive Science, Nagoya City University Graduate School of Medical Sciences, Nagoya, Aichi Japan

**Keywords:** Alzheimer’s disease, Transgenic mice, General anesthesia, Postanesthetic effects, Behavioral phenotype, App^NL−G−F/NL−G−F^

## Abstract

As the proportion of elderly in society increases, so do the number of older patients undergoing surgical procedures. This is concerning as exposure to anesthesia has been identified as a risk factor for Alzheimer’s disease (AD). However, the causal relationship between clinical AD development and anesthesia remains conjectural. Preclinical studies have demonstrated that anesthesia, such as halothane, isoflurane, and sevoflurane, induces AD-like pathophysiological changes and cognitive impairments in transgenic mouse models of AD. Desflurane does not have these effects and is expected to have more potential for use in elderly patients, yet little is known about its effects, especially on non-cognitive functions, such as motor and emotional functions. Thus, we examined the postanesthetic effects of desflurane and sevoflurane on motor and emotional function in aged *App*^*NL−G−F/NL−G−F*^ (App-KI) mice. This is a recently developed transgenic mouse model of AD exhibiting amyloid β peptide (Aβ) amyloidosis and a neuroinflammatory response in an age-dependent manner without non-physiological amyloid precursor protein (APP) overexpression. Mice were subjected to a short behavioral test battery consisting of an elevated plus maze, a balance beam test, and a tail suspension test seven days after exposure to 8.0% desflurane for 6 h or 2.8% sevoflurane for 2 h. App-KI mice showed significant increments in the percentage of entry and time spent in open arms in the elevated plus maze, increments in the number of slips and latency to traverse for the balance beam test, increments in the limb clasping score, increments in immobile duration, and decrements in latency to first immobile episode for the tail suspension test compared to age-matched wild type (WT) controls. Desflurane- and sevoflurane-exposed App-KI mice showed a delayed decrement in the number of slips for each trial in the balance beam test, while air-treated App-KI mice rapidly improved their performance, and increased their clasping behavior in the tail suspension test. Furthermore, App-KI inhibited the change in membrane GluA3 following exposure to anesthetics in the cerebellum. These results suggest high validity of App-KI mice as an animal model of AD.

**Supplementary Information:**

The online version contains supplementary material available at 10.1186/s13041-022-00910-1.

## Introduction

Alzheimer’s disease (AD) is the most common form of dementia and is characterized by extracellular accumulation of amyloid β peptide (Aβ) accompanied by neuroinflammation, an intracellular neurofibrillary tangle (NFT) caused by hyperphosphorylation of tau protein, and a loss of neuronal cells. Although the main clinical symptoms are memory impairments and cognitive deficits, associated non-cognitive motor function and emotional disturbances have also been reported [1–3].

Some retrospective cohort and case–control clinical studies have suggested that general anesthesia exposure associated with surgery is another risk factor for developing AD [4–7]. However, other studies show no link between anesthesia/surgery and AD, dementia, or cognitive dysfunctions [8–11]; therefore, causal links between anesthesia/surgery and developing AD remains conjectural [12–18]. As the proportion of elderly in society increases, the number of patients with AD and presumably presymptomatic AD patients undergoing surgery involving general anesthesia may also increase. Thus, elucidating the causal relationship between general anesthesia and the development of AD pathology is critical.

Genetically modified mice are one of the most practical tools available today for translational AD research, bridging clinical and basic research. Many transgenic AD mouse models carry familial AD (FAD) mutations in genes that encode amyloid precursor protein (APP), presenilin-1 (PS1), and presenilin-2 (PS2). PS1 and PS2 are the main components of γ-secretase that cleaves APP to generate Aβ fragments. FAD accounts for less than 5% of all AD; however, causative genes have been identified, and FAD shows morphologically similar Aβ amyloidosis and tauopathy with sporadic AD (SAD). Studies on FAD mutant mouse models are based on the widely accepted "amyloid cascade hypothesis," which states that an increase in Aβ in the brain causes excessive phosphorylation of tau protein and its intracellular deposition, resulting in neuronal death and cognitive dysfunction. Although SAD is multifactorial and has many pivotal points, it has been reported that beta-secretase activity that catalyzes the first stage cleavage of APP (β-cleavage) increased in SAD [19–22]. Increment in beta-secretase activity is also a characteristic of the FAD Swedish mutation introduced into many genetically modified AD mice. At present, mice with FAD mutations (except for mice that have directly modified tau protein) exhibit amyloid deposits, but are unable to reproduce NFT-like tau pathology [23–25]. In humans, Aβ accumulation precedes tau pathology by more than 10 years, and the short lifespan of mice (2–3 years) may explain why NFTs cannot be reproduced. In contrast, mice with FAD mutations exhibiting Aβ accumulation are thought to represent Aβ aggregate pathology in the presymptomatic early stages of AD. Thus, mice with FAD mutations provide a useful animal model for investigating the effects of anesthesia on presymptomatic individuals at high risk of developing AD with increased Aβ levels in the brain.

Studies have demonstrated that exposure to general anesthesia, such as halothane, isoflurane, and sevoflurane contributes to cognitive impairment and exacerbates AD-like pathophysiological symptoms in transgenic AD mouse models [26–32]. Nevertheless, desflurane, a newer inhaled halogenated ether, frequently used in clinical settings, has little effect on learning and memory in AD model mice. In fact, desflurane-treated 3xTgAD mice (i.e., triple transgenic mice overexpressing APP with FAD Swedish mutation, mutant PS1, and human Tau transgenes) did not have cognitive impairment compared to non-treated mice [33]. Similarly, desflurane did not affect learning and memory performance in the Barnes maze test for double transgenic mice carrying APP and PS1 transgenes with five FAD mutations (5xFAD mice) [34].

We recently reported a comprehensive behavioral analysis of the postanesthetic effects of desflurane in young adult mice [35]. Results showed no observed differences between the control and desflurane-treated groups, except for mild temporal effects on motor coordination in the balance beam test. In addition, desflurane-treated mice had a tendency to spend more time in open arms in the elevated plus maze, and to decrease latency for the first episode of immobility in tail suspension tests with a sufficiently large effect size. These results suggest that postanesthetic effects of desflurane mainly affect non-cognitive motor function, but may also affect emotional behavior. The effects of desflurane on these functions may be more pronounced in fragile old animals or transgenic AD model mice than in healthy young adults.

Another issue to consider is that all previous studies investigating postanesthetic effects on transgenic AD model mice used transgenic mouse strains that overexpressed APP. Overexpression of APP induces Aβ overproduction, causing insoluble Aβ aggregation, but simultaneously induces overproduction of other APP fragments with physiological effects unrelated to AD. *App*^*NL-G-F/NL-G-F*^ (App-KI) mice were developed to address this issue and show an increased Aβ42/40 ratio and Aβ deposition without non-physiological overexpression of APP. In App-KI mice, Aβ sequence was humanized and introduced three FAD mutations (i.e., Swedish, Beyreuther/Iberian, and Arctic) into the endogenous mouse APP gene [36]. Further, App-KI mice showed Aβ amyloidosis, synaptic alterations, and neuroinflammatory responses with slight memory impairment. Previous studies revealed various behavioral phenotypes in App-KI mice, such as mild deficits in learning and memory function [36–42], sociality [37, 43], and anxiety [37, 39, 40, 43]. However, there are no studies focusing on the overall motor function or the more specific effects of general anesthesia on motor function in App-KI mice.

In the current study, we focused on the effects of desflurane on motor function in aged App-KI mice and age-matched controls because of the potential benefits of using desflurane in elderly patients, specifically that it remains in the body for a shorter amount of time than other inhaled halogenated anesthetics [44]. Further, we also assessed the effects of sevoflurane since, like desflurane, it is an inhaled anesthetic frequently used in modern clinical settings. We performed a short battery of tests on mice exposed to desflurane and sevoflurane seven days prior to performing the tests. There were three behavioral tests: an elevated plus maze, a balance beam test, and a tail suspension test.

We found that desflurane and sevoflurane induced motor learning deficits specifically in App-KI mice. That is, anesthesia-exposed App-KI mice showed a delayed decrement in the number of slips for each trial in the balance beam test, while air-treated App-KI mice rapidly improved their performance. The cerebellum is important for non-declarative memory, such as motor learning [45–48], so we expected that anesthesia exposure in App-KI will induce pathophysiological changes in the cerebellum, leading to deficits in motor learning. To elucidate the biological basis underlying deficits in cerebellum-dependent behavior observed in anesthesia-exposed App-KI mice, we also investigated expression levels of α-amino-3-hydroxy-5-methyl-4-isoxazolepropionic acid (AMPA) receptor subunits (GluA1, 2, 3, and 4), controlling for cerebellum-dependent behaviors via modification of long-term potentiation (LTP) and long-term depression (LTD) [49, 50].

## Methods

### Animals

This study was carried out in strict accordance with the recommendations of the Guide for Care and Use of Laboratory Animals of Yokohama City University. The protocol was approved by the Committee on the Ethics of Animal Experiments of Yokohama City University [F-A-18-006, F-D-20-4].

App-KI mice harboring Swedish and Beyreuther/Iberian mutations with the Arctic mutation in APP gene mice [36] and its wild type (C57BL/6J: WT) were used. The original lines of App-KI mice were obtained from the RIKEN Center for Brain Science (Wako, Japan). The genotype was verified from tail biopsies using polymerase chain reaction (PCR). The detailed protocol is described in Saito et al. [36]. The following primers were used: 5′-ATCTCGGAAGTGAAGATG-3′, 5′-ATCTCGGAAGTGAATCTA-3′, 5′-TGTAGATGAGAACTTAAC-3′ and 5′-CGTATAATGTATGCTATACGAAG-3′. App-KI and WT mice were housed in separate cages after 4 weeks of age. Mice were housed in groups of three to five per cage. Throughout the experiment, the animals were maintained in a temperature-controlled room (23 ± 2 °C) with a 14-h light 10-h dark cycle (light period, 5:00 AM to 7:00 PM) and provided food and water ad libitum.

### General anesthesia

When the mice reached 24 months old, WT and App-KI mice were randomly assigned to either the control group (WT-Air: n = 7, App-Air: n = 14), desflurane exposure group (WT-Des: n = 8, App-Des: n = 12), or sevoflurane exposure group (WT-Sev: n = 6, App-Sev: n = 12). General anesthesia exposure was performed using methods from our previous study [35]. Briefly, mice receiving general anesthesia were placed in a translucent plastic chamber (25.0 × 17.5 × 8.0 cm) within a thermostatic bath (34 ± 2 °C). The chamber was maintained with oxygen and nitrogen (FiO_2_ = 0.33) at 6 l/min. The concentrations of desflurane and sevoflurane were maintained at 8.0% for 6 h and 2.8% for 2 h, respectively, corresponding to a minimum alveolar concentration (MAC) of 1.3. Carbon dioxide in the chamber was maintained below 3 mm Hg. The gases were monitored using a Capnomac ULTIMA monitor (Datex, Helsinki, Finland). Desflurane has lower solubility in blood and body tissue, and has lower metabolism than other inhaled halogenated anesthetics, indicating that it remains in the body for a shorter time than other inhaled halogenated anesthetics [44]. Specifically, when exposed for 2 h, sevoflurane took about 1.5 days for 99.9% brain elimination, while desflurane took less than one day. After six hours of desflurane exposure, it took 1.5 days for 99.9% brain elimination [51]. To investigate the long-term post-anesthetic effects of desflurane compared to 2 h exposure to sevoflurane, we determined that a 6 h exposure to desflurane was appropriate. After exposure, the animals returned to their cages and fully recovered within 15 min. In the control group, one mouse at a time was placed in a plastic chamber flushed with the same carrier gas for 5 min, approximately the time necessary for the mouse to lose consciousness under anesthesia, and then returned to its original cage.

It is important that different types of anesthesia with different exposure durations have similar effects on sedation and the physiological state of the circulatory system; therefore, we conducted additional experiments assessing the effects of sevoflurane and desflurane on time to recovery of consciousness and blood gases analysis using separate groups of mice. Due to the limitations of life resources, we repeatedly exposed the same mice (WT: n = 4, App-KI: n = 3) to air, sevoflurane, and desflurane at 7-day intervals in the same manner as described above, and collected blood from the tail artery at the end of each exposure. Blood gas analysis was performed immediately after sample collection using an i-STAT 1 analyzer with a CG8 + cartridge (Abbot Point of Care, Princeton, NJ, USA) to confirm the values of pH, partial pressure of arterial carbon dioxide, partial pressure of arterial oxygen, base excess in the extracellular fluid compartment, arterial bicarbonate, arterial total carbon dioxide, arterial oxygen saturation, arterial sodium, arterial potassium, arterial ionized calcium, arterial glucose, arterial hematocrit (% packed cell volume), and arterial hemoglobin. Time to recovery of consciousness for each anesthesia was also measured as latency to spontaneous movement.

### Behavioral tests

**Fig. 1:**
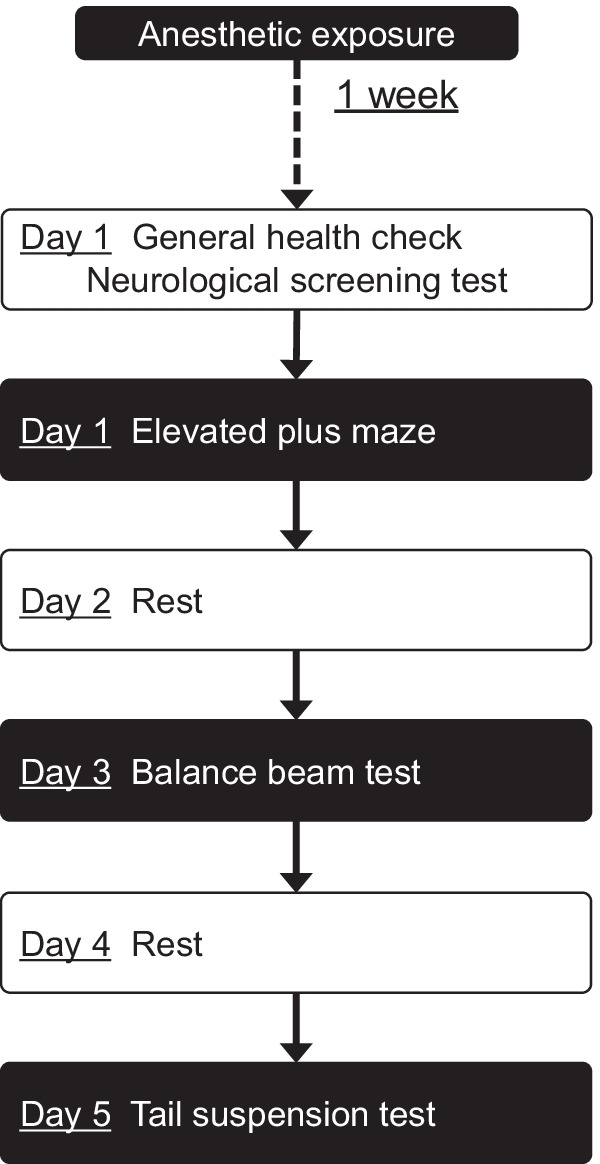
Timeline of the series of behavioral tests. One week after exposure to general anesthesia, the behavioral tests were serially carried out. After general health check and neurological screening tests, mice were examined an elevated plus maze on day 1. After one day of rest to avoid the carry-over effect, a balance beam test was performed on day 3 and as well, a tail suspension test was performed on day 5

One week after exposure to general anesthesia, behavioral tests were serially carried out to assess the post-anesthetic effects of desflurane and sevoflurane on the emotional and motor functions of aged App-KI mice, as outlined in Fig. 1. All behavioral tests were performed and blindly measured by a trained observer. Prior to testing, each mouse was observed in a clean cage for a general health check and neurological screening tests based on methods from our previous reports [35, 52–54]. The neurological screening tests were designed to detect any gross abnormalities in physical function. The ear-twitch reflex occurred when the pinna was touched with a cotton swab from behind, resulting in immediate movement of the ear. The eye-blink reflex occurred when a cotton swab approached the eye, resulting in blinking. The whisker-touch reflex was tested by lightly touching the whiskers of a freely moving mouse. Normal mice stop moving their whiskers and turn their head to the side where the whiskers are being touched. The postural reflex was evaluated by placing the mouse in an empty cage and shaking the cage, eliciting the extension of all four legs to maintain an upright, balanced position. The righting reflex was tested by turning the mouse over onto its back, eliciting an immediate turnover response restoring upright posture on all four feet.

### Elevated plus maze

To evaluate state anxiety-like behavior for open spaces and heights, an elevated plus maze was conducted on Day 1. The apparatus consists of two open arms (297 × 54 mm) and two closed arms (300 × 60 × 150 mm) extending from a common central platform (60 × 60 mm). A small raised lip (3 mm) around the perimeter of the open arms prevented the mouse from falling. The apparatus was constructed from polypropylene, with gray floor and gray walls, and elevated 40 cm above the floor. Mice were placed individually on the center square facing an open arm and allowed to freely explore the apparatus under overhead fluorescent lighting (200 lx) for 5 min. Time spent in the open arms and open and closed arm entries (all four paws in an arm) were scored by a highly trained observer using behavioral scoring software (ANY-maze, Stoelting, IL, USA).

### Balance beam test

Motor coordination and balance were assessed by measuring the mice’s ability to traverse a graded series of narrow beams to reach an enclosed safety platform on Day 3. The beams consisted of a long square strip of metal (1 m in length) with a cross-section of 12-mm. The beams were placed horizontally 50 cm above the bench surface, with one end mounted on a narrow support and the other end attached to an enclosed box (20 cm^2^) into which the mouse could escape. Lights (1200 lx) were positioned above and on one side of the start of the beam. The test was composed of six trials separated by 30 min inter-trial intervals. In each trial, the mice were placed at the start of the beam and allowed to traverse the beam to the enclosed box. The cut-off time for each trial was 60 s. The number of times the hind feet slipped off each beam and the latency to traverse each beam were recorded for each trial.

### Tail suspension test

Postural reflex and coping behavior in a hopeless situation (antidepressant-like activity) were assessed using a tail suspension test on Day 5. Mice were securely fastened to a flat metallic surface by the tip of the tail (2–3 cm) using medical adhesive tape and suspended 30 cm above the ground in a 40 cm^3^ white plexiglass box that isolated the mouse from visual distractions. The latency to first immobility, defined as the absence of limb movement and the time to immobility were sampled using the ANY-maze. Limb clasping behavior during the first 30 s was monitored by a trained experimenter and was scored on a scale from zero to four: 0 = no clasping behavior, 1 = one hind limb was retracted and the toes were splayed, 2 = both hind limbs were retracted and toes were splayed, 3 = both hind limbs were retracted and toes were clasping, 4 = clasping of all paws.

### Western blotting

Seven days after the behavioral tests, some mice (WT-Air: n = 3, App-Air: n = 5, WT-Des: n = 3, App-Des: n = 5, WT-Sev: n = 3, App-Sev: n = 5) were decapitated with a guillotine under anesthesia by a well-trained experimenter. Brains were promptly removed, washed in ice-cold phosphate-buffered saline (PBS), and stored at − 80 °C until processing. The cerebellar tissue was dissected out and homogenized in 6 volumes of ice-cold 0.32 M sucrose PBS containing a protease inhibitor cocktail (Nacalai Tesque, Kyoto, Japan) and phosphatase inhibitor cocktail (Nacalai Tesque) using a disposable polypropylene homogenizer (450 rpm, 50 strokes; BioMasher II, Nippi, Tokyo, Japan). The homogenized tissue was divided into two portions at a ratio of 1:6 for crude fraction extraction (1/7 of total volume) and crude membrane fraction extraction (6/7 of total volume). For crude fraction extraction, 0.1 M EGTA (5 μl/ml), 0.5 M EDTA (1 μl/ml) and 20% TritonX (25 μl/ml) were added to the former homogenate, sonicated 10 s on ice, incubated 30 min on ice, and centrifuged at 17,300×*g* for 15 min at 4 °C. The supernatant was collected as a crude fraction. For crude membrane fraction extraction, the latter homogenate was centrifuged at 1400×*g* for 10 min at 4 °C. The supernatant was centrifuged at 13,800×*g* for 10 min at 4 °C to obtain a crude membrane fraction (P2). To wash the P2 fraction, the pellet was resuspended in 1 ml ice-cold 0.32 M sucrose PBS containing a protease inhibitor cocktail and phosphatase inhibitor cocktail, centrifuged at 17,300×*g* for 5 min at 4 °C, and the supernatant was discarded. This wash procedure was repeated four times. Afterward, the pellet was resuspended in twice the original cerebellar tissue volumes of PBS containing 0.1 M EGTA (5 μl/ml), 0.5 M EDTA (1 μl/ml), 20% TritonX (25 μl/ml), a protease inhibitor cocktail and phosphatase inhibitor cocktail, mixed by inversion for 60 min at 4 °C, and centrifuged at 17,300×*g* for 10 min at 4 °C. The supernatant was collected as a crude membrane fraction. The protein concentrations for both the crude and crude membrane fraction supernatants were determined using the Pierce bicinchoninic acid protein assay kit (Thermo Fisher Scientific, Waltham, MA, USA). The supernatants were sampled in a SDS sample buffer and adjusted to a similar protein concentration for all samples. Samples were boiled for 5 min at 95 °C to denature the protein and subjected to 7.5% SDS-PAGE followed by electrotransfer to a polyvinylidene difluoride membrane. The membranes were blocked for 1 h with 2% ECL blocking agent (GE Healthcare, Chicago, IL, USA) in Tris buffer saline containing 0.1% tween 20 (TBS-T) and incubated overnight at 4 °C with anti-GluA1 (1:1000; #04-855, Sigma-Aldrich, St. Louis, MO, USA), anti-GluA2 (1:1000; #ab20673, Abcam, Cambridge, UK), anti-GluA3 (1:1000; #5117, Cell Signaling Technology, Danvers, MA, USA), anti-GluA4 (1:1000; #23350-1-AP, Proteintech, Rosemont, IL, USA), anti-β-actin (1:10000; #A5441, Sigma-Aldrich), and anti-HSP60 (1:10000; #ab45134, Abcam) antibodies. The blocking agent was used to prepare all antibody reagents. After washing with TBS-T (10 min × 3), membranes were incubated for 1 h at room temperature with secondary antibodies (1:10000; #175–6515, #170-6520, BioRad, Hercules, CA, USA). After washing with TBS-T (10 min × 3), protein visualization was conducted using the ECL detection system (#RPN2235, #RPN 2236, GE Healthcare). The membranes were scanned using a fluorescence imaging system (EZ-Capture MG; ATTO, Tokyo, Japan), and the band densities were measured using ImageJ ver.1.53c [55, 56]. The protein expression levels for crude and crude membrane fractions were normalized to β-actin and HSP60 levels, respectively. Individual values were normalized to the mean values of the WT-Air group (presented as 1).

### Statistical analysis

Data for body weight, body size, percentage of open arm entries and time spent in the open arms for the elevated plus maze, total immobile duration and latency to first immobility in the tail suspension test, and western blotting were analyzed using a two-way analysis of variance (ANOVA) (gene × anesthesia). Data from the balance beam test and distance traveled in the open and closed arms for the elevated plus maze test were analyzed using a three-way mixed design ANOVA (balance beam test: gene × anesthesia × trial; elevated plus maze test: gene × anesthesia × arms), followed by a post-hoc analysis adjusted by Shaffer's modified sequentially rejective Bonferroni procedure. Data from the general health check, with the exception of body weight, body size, neurological screening tests, and limb clasping in the tail suspension test, were analyzed using a Kruskal–Wallis H test followed by a post-hoc analysis using the Mann–Whitney U test adjusted by Bonferroni correction. Data from the blood gas analysis and latency to spontaneous movement once anesthesia exposure ended were analyzed using a two-way mixed design ANOVA (gene × anesthesia).

## Results

### General health

The general health check showed no statistically significant differences among all groups [*p* > 0.050], except for the bald patch [*χ*^*2*^ (5) = 13.168, *p* = 0.022] (Table 1). A post hoc analysis revealed that App-KI mice had fewer bald patches than WT control mice [*p* = 0.001]. The neurological reflex performance for all groups was similar [p > 0.050] (Table 1). Additional experiments assessing the effect of each anesthesia type on the physiological state of the circulatory system once anesthesia exposure ended revealed that there were no statistically significant differences in blood gas components among all groups [*p* > 0.050] (Table 2). Further, all groups showed similar latency to spontaneous movement from anesthesia (WT-Des, 162.0 ± 26.4 s; WT-Sev, 144.5 ± 16.5 s; App-Des, 185.0 ± 40.31 s; App-Sev, 167.0 ± 7.76 s; Values are mean ± SEM) [*p* > 0.050], suggesting neither type of anesthesia affected the physiological state of the circulatory system.

**Table 1:** General health and neurological reflexes

Genotype	WT			App-KI		
Anesthesia	Air	Desflurane	Sevoflurane	Air	Desflurane	Sevoflurane
General health check						
Body weight (g)	33.0 ± 0.9	31.1 ± 0.8	30.3 ± 1.4	31.2 ± 0.6	30.6 ± 0.6	30.7 ± 0.9
Body size (% with average size)	106.2 ± 2.8	100.1 ± 2.6	97.6 ± 4.5	100.4 ± 2.0	98.4 ± 2.0	98.7 ± 2.8
Bald patch (% with)	28.6	25.0	50.0	0.0	8.3	0.0
Fur (% with normal fur)	100.0	62.5	50.0	85.7	83.3	100.0
Crustiness around the nostrils/eyes (% with)	0.0	0.0	0.0	0.0	0.0	0.0
Ear pinna/Footpad color (% with normal color)	100.0	100.0	100.0	100.0	100.0	100.0
Leisons on the feet/tail (% with)	14.3	0.0	0.0	0.0	0.0	0.0
Scabs on the tail, rump, back (% with)	0.0	0.0	0.0	0.0	0.0	0.0
Tumor (% with)	0.0	0.0	0.0	0.0	0.0	0.0
Gait (% with normal gate)	100.0	100.0	100.0	100.0	100.0	100.0
Posture (% with normal posture)	100.0	100.0	100.0	100.0	100.0	100.0
Whisker (% with normal whisker)	42.9	50.0	66.7	71.4	83.3	75.0
Neurological screening test						
Ear twich (% with quick response)	100.0	100.0	100.0	100.0	100.0	100.0
Eye blink (% with normal response)	85.7	100.0	100.0	85.7	75.0	91.7
Whisker-touch (% with normal response)	42.9	50.0	66.7	71.4	75.0	75.0
Postual reflex (% with normal response)	100.0	100.0	100.0	100.0	100.0	91.7
Righting reflex (% with normal response)	100.0	100.0	100.0	100.0	100.0	100.0

The general health check indicated that there are no differences among each group [ps > 0.050], except bald patch [χ^2^ (5) = 13.168, p = 0.022]. A post hoc analysis revealed that App-KI mice showed fewer bald patch than WT control [p = 0.001]. The neurological reflexes of all groups showed similar performances [ps > 0.050]. (WT-Air: N = 7, WT-Des: N = 8, WT-Sev: N = 6, App-Air: N = 14, App-Des: N = 12, App-Sev: N = 12). Values are mean ± SEM

**Table 2:** Arterial blood gas analysis

Genotype	WT			App-KI		
Anesthesia	Air	Desflurane	Sevoflurane	Air	Desflurane	Sevoflurane
Measurement						
pH	7.44 ± 0.01	7.41 ± 0.02	7.47 ± 0.03	7.47 ± 0.03	7.38 ± 0.00	7.46 ± 0.01
pCO_2_ (mmHg)	32.28 ± 1.34	36.20 ± 2.06	33.58 ± 3.17	34.43 ± 3.58	38.05 ± 0.04	30.47 ± 0.91
pO_2_ (mmHg)	87.75 ± 3.38	91.25 ± 10.18	124.50 ± 19.61	86.00 ± 1.25	85.50 ± 1.77	98.00 ± 4.97
BEecf (mmol/l)	− 2.50 ± 1.15	− 2.00 ± 1.06	0.25 ± 0.65	0.67 ± 0.72	− 3.00 ± 0.00	− 2.33 ± 0.72
${\text{HCO}}_{3}^{ - }$ (mmol/l)	21.73±1.04	22.83 ± 0.82	23.80 ± 0.93	24.50 ± 1.11	22.45 ± 0.04	21.70 ± 0.74
TCO_2_ (mmol/l)	22.50 ± 1.15	24.00 ± 1.06	25.00 ± 0.94	25.33 ± 1.19	24.00 ± 0.00	22.67 ± 0.72
sO_2_ (%)	97.25 ± 0.41	96.25 ± 1.08	98.50 ± 0.75	97.00 ± 0.47	96.50 ± 0.35	98.00 ± 0.47
Na (mmol/l)	144.50 ± 0.83	139.75 ± 1.60	143.25 ± 1.08	145.67 ± 1.52	143.50 ± 1.06	140.67 ± 1.09
K (mmol/l)	4.23 ± 0.16	4.38 ± 0.18	4.63 ± 0.26	4.87 ± 0.12	4.20 ± 0.21	4.17 ± 0.07
iCa (mmol/l)	1.08 ± 0.03	1.07 ± 0.05	1.03 ± 0.04	1.12 ± 0.04	1.12 ± 0.02	0.94 ± 0.03
Glu (mg/dl)	170.50 ± 23.98	194.75 ± 8.22	165.75 ± 6.79	188.33 ± 15.49	170.00 ± 14.85	142.33 ± 13.02
Hct (%PCV)	35.50 ± 1.03	34.75 ± 1.52	33.50 ± 1.44	38.33 ± 1.09	39.00 ± 0.71	33.67 ± 0.27
Hb (g/dl)	12.08 ± 0.35	11.80 ± 0.53	11.43 ± 0.49	13.03 ± 0.35	13.25 ± 0.25	11.47 ± 0.11

The analysis of artial blood gases indicated that there are no differences among each group [ps > 0.050]. (WT-Air: N = 4, WT-Des: N = 4, WT-Sev: N = 4, App-Air: N = 3, App-Des: N = 3, App-Sev: N = 3). Values are mean ± SEM

*pCO*_*2*_ partial pressure of arterial carbon dioxide, *pO*_*2*_ partial pressure of arterial oxygen, *BEecf* base excess in the extracellular fluid compartment, ${\text{HCO}}_{3}^{ - }$ arterial bicarbonate, *TCO*_*2*_ arterial total carbon dioxide, *sO*_*2*_ arterial oxygen saturation, *iCa* arterial ionized calcium, *Glu* arterial glucose, *Hct* (%PCV), arterial hematocrit (% packed cell volume), *Hb* arterial Hemoglobin

### Elevated plus maze

**Fig. 2:**
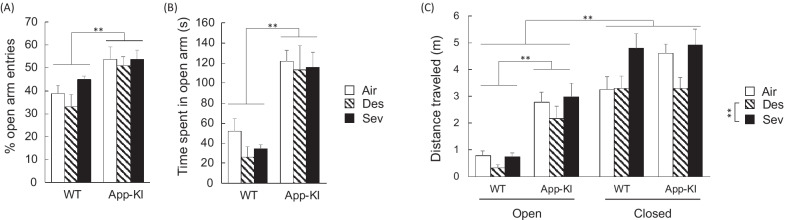
Effect of general anesthesia on aged App-KI mice in an elevated plus maze. Mean (+ SEM) percentage of open arm entries out of total arm entries (**A**), time spent in open arm (**B**) and traveled distance in open arms and closed arms (**C**) in each group, WT-Air (n = 7), WT-Des (n = 8), WT-Sev (n = 6), App-Air (n = 14), App-Des (n = 12) and App-Sev (n = 12) group, are shown. **p < 0.010

The state anxiety-like behavior of mice was assessed by measuring the percentage of open arm entries out of total arm entries and time spent in the open arms. The mean percentage of open arm entries out of total arm entries in the elevated plus maze was higher in the App-KI group than in the WT group [main effect of gene: F (1, 53) = 17.017, *p* < 0.001, partial η^2^ = 0.243] (Fig. 2A). The percentage of open arm entries was not affected by general anesthesia exposure [main effect of anesthesia: F (2, 53) = 1.657, *p* = 0.200, partial η^2^ = 0.059], and there was no interaction between gene and anesthesia [interaction: F (2, 53) = 0.609, *p* = 0.548, partial η^2^ = 0.022]. For the mean time spent in the open arm, the same tendency was observed for the percentage of open arm entries [main effect of gene: F (1, 53) = 28.159, *p* < 0.001, partial η^2^ = 0.347; main effect of anesthesia: F (2, 53) = 0.493, *p* = 0.614, partial η^2^ = 0.018; interaction: F (2, 53) = 0.135, *p* = 0.874, partial η^2^ = 0.005] (Fig. 2B). The mean distance traveled in the open and closed arms for the elevated plus maze test is shown in Fig. 2C. A three-way ANOVA revealed significant main effects for all three factors: gene [F (1, 53) = 21.671, *p* < 0.001, partial η^2^ = 0.290], anesthesia [F (2, 53) = 5.473, *p* = 0.007, partial η^2^ = 0.170], and arms [F (1, 53) = 54.683, *p* < 0.001, partial η^2^ = 0.508], whereas interactions among these factors were not statistically significant [*p*s > 0.050] with the exception of the interaction between gene and arms [F (1, 53) = 5.609, *p* = 0.022, partial η^2^ = 0.096]. A post hoc analysis revealed that the desflurane-exposed group moved shorter distances than the sevoflurane-exposed group [*p* = 0.005], but these two anesthesia exposure groups did not show statistically significant differences compared to the air-exposed group [*p* > 0.050]. Further analysis indicated that this type of anesthesia effect on locomotor activity was only observed in the App-KI and closed arms groups, indicating that desflurane affected spontaneous locomotor activity and drive for exploration to a safe zone, rather than anxiety-like behavior of aged mice. Further, App-KI mice moved longer than WT controls in open arms [*p* < 0.001], while there was no statistically significant difference between the two groups in closed arms [*p* > 0.050]. These results suggest that aged App-KI mice exhibited excessive anxiolytic-like behavior and that neither desflurane nor sevoflurane affected these performances.

### Balance beam test

**Fig. 3:**
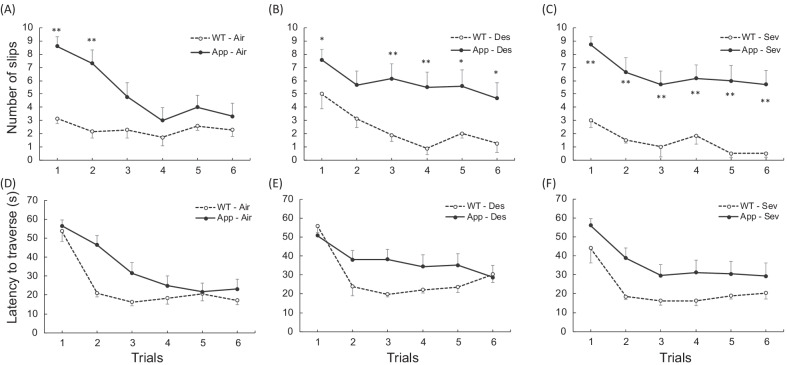
Effect of general anesthesia on aged App-KI mice in a balance beam test. Mean (+ SEM) number of times the hind feet slipped off in each trial (**A**–**C**) and latency to traverse (**D**–**F**) in each group, WT-Air (n = 7), App-Air (n = 14), WT-Des (n = 8), App-Des (n = 12), WT-Sev (n = 6) and App-Sev (n = 11) group, are shown. *p < 0.050; **p < 0.010 compared to corresponding WT group

Motor coordination and balance were assessed by measuring the number of times the hind feet slipped off and latency to traverse for each trial of the balance beam test. The mean number of times the hind feet slipped off in each group is shown in Fig. 3A–C. A three-way repeated ANOVA revealed that the mean number of slips was larger in the App-KI group than in the WT group [main effect of gene: F (1, 51) = 32.507, *p* < 0.001, partial η^2^ = 0.389], and decreased through the trials [main effect of trial: F (5, 255) = 14.328, *p* < 0.001, partial η^2^ = 0.219]. General anesthetics did not affect the number of slips [main effect of anesthesia: F (2, 51) = 0.095, *p* = 0.909, partial η^2^ = 0.004]. Although the interactions, gene × anesthesia, gene × trial, and anesthesia × trial were not statistically significant [*p* > 0.050], there was a statistically significant interaction between these three factors [F (10, 255) = 2.048, *p* = 0.041, partial η^2^ = 0.074]. A post-hoc analysis adjusted by Shaffer's modified sequentially rejective Bonferroni procedure revealed that when compared within the App-KI or WT group, there were no statistically significant differences among the air-, sevoflurane-, and desflurane-treated groups for each trial [*p*s > 0.050]. Further, the number of slips in the 1st and 2nd trial for the air-treated App-KI group were larger than those after the third trial [*p*s < 0.050], suggesting that motor learning occurred in the App-KI group when not exposed to anesthesia. On the other hand, in both App-KI groups exposed to anesthesia, the number of slips did not decrease with each trial [*p*s > 0.050]. In all of the trials, except for the 2nd trial of desflurane exposure groups, anesthesia-exposed App-KI groups exhibited more slips than the anesthesia-exposed WT groups [*p*s < 0.050], whereas the air-treated App-KI group showed more slips than the air-treated WT group only in the 1st and 2nd trial [*p* < 0.010], suggesting that both desflurane and sevoflurane induced motor learning deficits in App-KI mice.

The mean latency to traverse was longer in the App-KI group than in the WT group [main effect of gene: F (1, 51) = 8.271, *p* = 0.006, partial η^2^ = 0.140] (Fig. 3D–F). Similar to the number of slips, the main effect of trial was statistically significant [F (5, 255) = 46.216, *p* < 0.001, partial η^2^ = 0.475], and the main effect of anesthesia was not statistically significant [F (5, 51) = 0.625, *p* < 0.539, partial η^2^ = 0.024]. There was a statistically significant interaction between gene and trial [F (5, 255) = 3.945, *p* = 0.007, partial η^2^ = 0.072], but other interactions were not statistically significant [*p* > 0.050]. A post hoc analysis revealed that latency to traverse in the App-KI group was longer in the 1st and 2nd trials compared to the 3rd, 4th, 5th and 6th trials, while latency in the WT group was longer in the 1st trial than in the other trials. Further, the App-KI group showed longer latency than the WT group in the 2nd, 3rd, and 4th trials, while there were no statistically significant differences in the 1st, 5th, and 6th trials, suggesting delayed motor learning in App-KI mice.

### Tail suspension test

**Fig. 4:**
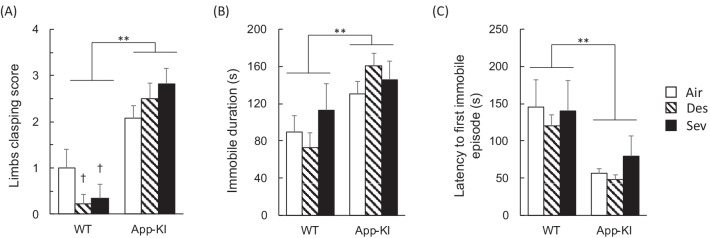
Effect of general anesthesia on aged App-KI mice in a tail suspension test. Mean (+ SEM) limbs clasping score (**A**), immobile duration (**B**) and latency to first immobile episode (**C**) in each group, WT-Air (n = 7), WT-Des (n = 8), WT-Sev (n = 6), App-Air (n = 13), App-Des (n = 11), and App-Sev (n = 11) group, are shown. **p < 0.010, †p < 0.010 compare to corresponding anesthesia-treated App-KI group

To assess postural reflex and antidepressant-like activity, the limb clasping score, total immobile duration, and latency to the first immobile episode were measured during a tail suspension test. The Kruskal–Wallis test revealed statistically significant differences [χ^2^ (5) = 28.24, *p* < 0.001] in the limb clasping score (Fig. 4A). A post hoc analysis revealed that the App-KI group exhibited significantly higher limb clasping scores than the WT group [U = 100, *p* < 0.001], whereas anesthesia did not affect the limb clasping score [χ^2^ (2) = 0.44, *p* = 0.804]. Further, the Mann–Whitney U test adjusted by Bonferroni correction revealed that the anesthesia-treated App-KI group showed higher clasping scores than the WT group [desflurane: U = 12.50, p = 0.002; sevoflurane: U = 3.50, *p* = 0.006], whereas the air-treated App-KI group did not differ from the WT group [U = 22.00, *p* = 0.163]. The mean immobile duration for the tail suspension test was longer in the App-KI group than in the WT group [main effect of gene: F (1, 50) = 11.773, *p* < 0.001, partial η^2^ = 0.191] (Fig. 4B). Immobility duration was not affected by general anesthesia exposure [main effect of anesthesia: F (2, 50) = 0.508, *p* = 0.605, partial η^2^ = 0.020], and there was no interaction between gene and anesthesia [interaction: F (2, 50) = 1.210, *p* = 0.307, partial η^2^ = 0.046].

For mean latency to first immobile episode in the tail suspension test, the same tendency as the immobile duration was observed [main effect of gene: F (1, 50) = 14.499, *p* < 0.001, partial η^2^ = 0.225; main effect of anesthesia: F (2, 50) = 0.620, *p* = 0.542, partial η^2^ = 0.024; interaction: F (2, 50) = 0.160, *p* = 0.852, partial η^2^ = 0.006] (Fig. 4C). These results suggest that aged App-KI mice exhibited deficits in posture reflex and shifted earlier from active coping behavior to passive coping behavior. Desflurane and sevoflurane did not affect these behaviors, except for hind limb clasping behavior.

### Western blotting

**Fig. 5:**
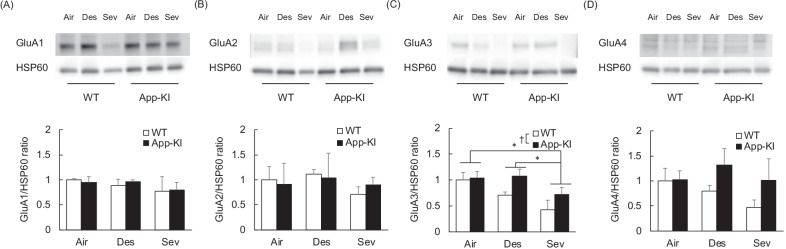
Effect of general anesthesia on membrane expression levels of AMPA receptors in the cerebellum in aged App-KI mice. Mean (+ SEM) protein expression ratio (normalized with the HSP60 expression levels) of GluA1 (**A**), GluA2 (**B**), GluA3 (**C**) and GluA4 (**D**) subunits in the crude membrane fractions in each group, WT-Air (n = 3), WT-Des (n = 3), WT-Sev (n = 3), App-Air (n = 5), App-Des (n = 5) and App-Sev (n = 5) group, are shown. Individual values were normalized with the mean values of WT-Air group as 1. *p < 0.050; ^†^p < 0.100

To elucidate the mechanism underlying the effects of anesthesia on the behavior of pre-symptomatic AD model mice, we assessed the expression levels of AMPA receptors in the cerebellum because AMPA receptors have been known to control cerebellum-dependent behaviors, including the balance beam test [49, 50]. AMPA receptor subunits in the crude fraction showed no difference in expression between App-KI and WT, or among anesthesia treatments [Additional file 1: Fig. S1; *p*s > 0.050], suggesting that the accumulation of Aβ and anesthesia exposure had no effect on the total AMPA receptor expression in the aged cerebellum. In contrast, the App-KI group showed higher tendencies for membrane expression of GluA3 compared with that of the WT group [F (1, 18) = 3.858, *p* = 0.065, partial η^2^ = 0.177] and a statistically significant effect of general anesthesia exposure [F (2, 18) = 4.978, *p* = 0.002, partial η^2^ = 0.356], while membrane GluA1, GluA2, and GluA4 showed no difference between App-KI and WT or among anesthesia treatments [*p* > 0.050] (Fig. 5). A post hoc analysis revealed that the sevoflurane-treated group had lower GluA3 expression levels than the other groups. The interaction between gene and anesthesia was not statistically significant [interaction: F (2, 18) = 0.729, *p* = 0.496, partial η^2^ = 0.075]. These results suggest that anesthesia suppresses membrane trafficking of GluA3 in aged mice and that the accumulation of Aβ may interfere with the inhibitory effect of anesthesia.

## Discussion

In the current study, we assessed the effects of two types of inhalation anesthetic used in modern clinical settings, sevoflurane and desflurane, on anxiety, motor function, and depression in aged App-KI mice as a model for early stage AD. Compared to age-matched WT controls, aged App-KI mice showed significant incremental increases in the percentage of entry, distance traveled, and time spent in open arms in the elevated plus maze, the number of slips and latency to traverse in the balance beam test, the limb clasping score, immobile duration, and decreases in latency to first immobile episode in the tail suspension tests. Neither desflurane nor sevoflurane affected the behavioral performance of App-KI and WT mice with the exception of motor learning performance of App-KI mice in the balance beam test. To elucidate the underlying mechanisms of anesthesia-induced motor learning deficits in App-KI mice, we examined the protein expression of AMPA receptors in the cerebellum. Age-matched WT mice showed a reduction in membrane GluA3 upon exposure to anesthetics, especially sevoflurane; however, accumulation of Aβ interfered with this inhibitory effect.

In the elevated plus maze, App-KI mice showed increased anxiolytic-like behavior compared to WT mice. Our results are in line with previous studies where App-KI that were 3, 6–10, and 15–18 months [37, 40] exhibited anxiolytic-like behavior in the elevated plus maze. This was also observed in several other APP overexpressing AD animal models [57–59], suggesting that the anxiolytic-like performance in these animals is associated with Aβ pathology rather than APP overexpression. Conversely, in the present study, neither anesthetic significantly affected anxiety-like behavior. A recent study reported that there is a distinct population of GABAergic neurons activated by anesthesia located in the capsular part of the central amygdala [60]. Optogenetic activation of this neuronal cluster induced inhibition of responses to the multimodal noxious stimulus, and their inactivation induced excessive responses to the stimulus. However, neither activation nor inactivation of this anesthesia-active neuron affected anxiety-like performances in the open field test or elevated plus maze. Our findings that anesthesia did not affect anxiety-like behavior in the elevated plus maze are consistent with these results. Our findings showed that sevoflurane tended to increase closed-arm locomotor activity, whereas desflurane did not. Moreover, desflurane-treated App-KI mice showed an inhibited level of locomotor activity in the closed arm at the same level as the WT control. Desflurane and sevoflurane might differentially affect brain regions, such as the periaqueductal gray, which integrates emotional and autonomic inputs with sensory information to produce appropriate behavioral and autonomic output.

Motor dysfunction is an important phenotype of early AD that precedes classical cognitive impairment [1, 61, 62]. The present results show that motor coordination in the balance beam test was impaired in App-KI mice, which is consistent with the fact that App-KI mice are an animal model mimicking features of pre-clinical AD with mild decline in cognitive function accompanied by Aβ accumulation [36, 63]. Most currently available transgenic mouse models of AD, and human AD patients, show progressive motor impairment with age [64–66]. Several studies have identified that motor impairment in APP mutants is due to axonopathy in spinal cord motor neurons [67–69]. However, all APP mutants used in these studies showed non-physiological overexpression of APP. The overexpression of APP results in an axonal transport traffic jam [70] because APP is associated with kinesin, a motor protein responsible for anterograde axonal transport, via JIP-1 [71]. Such axonal blockage might cause intracellular accumulation of APP, rather than extracellular accumulation of Aβ, resulting in axonal swelling, suggesting that deficits in axonal transport due to overproduction of APP can cause motor impairment [72]. Since the App-KI mice showed Aβ accumulation without non-physiological APP overexpression, the motor dysfunction observed in this study may have been derived from Aβ amyloidosis. However, the effect of an overproduced C-terminal fragment β of APP on motor impairment cannot be ruled out, because of the increment in cleavage of APP by β-secretase derived from the Swedish mutation.

In the present study, we demonstrated impairment in the postural reflex of App-KI mice in a tail suspension test. Although deficits in postural reflex have been previously reported in Tg2576 mice [73] and TgCRND8 mice [74], the current findings are the first report of postural reflex impairment in AD model mice without APP overexpression. A longer immobile duration and shorter immobile latency were observed in App-KI mice compared to WT mice. These results are typically interpreted as an increase in depression-like behavior, but this is the first study to demonstrate depression-like behavior in AD model mice without APP overexpression. However, the possibility that a motor deficit may contribute to depressive-like behavior also should be carefully considered.

We also demonstrated in the current study that both desflurane- and sevoflurane-exposed App-KI mice showed a delayed decrement in the number of slips for each trial in the balance beam test, while air-treated App-KI mice rapidly improved their performance. Moreover, anesthesia-exposed App-KI mice showed a high clasping score, which is typically interpreted as an index of cerebellar ataxia, exhibiting as motor learning deficits. The cerebellum is important for procedural memory [45–48], so we expected that anesthesia exposure in App-KI would induce pathophysiological changes in the cerebellum, leading to deficits in motor learning. In the AD animal model, APP/PS1 mice and TgCRND8 mice at the preplaque stage showed motor deficits, but not a motor learning deficit (i.e., deficit in performance improvement for each trial as shown in the current study), and a decline in LTD in cerebellar parallel fiber-Purkinje cell synapses, suggesting impairment in the cell biological basis of short-term motor learning [72, 75]. Further, the cerebellum, especially Purkinje cells, is vulnerable to various types of toxic damage [76, 77], including general anesthesia, such as sevoflurane [78] and desflurane [79].

To elucidate the biological basis underlying deficits in cerebellum-dependent behavior observed in anesthesia-exposed App-KI mice, we investigated the expression levels of AMPA receptor subunits in the cerebellum that have been known to control cerebellum-dependent behaviors, including the balance beam test. AMPA receptors control synaptic strength dynamically via membrane trafficking (LTP) and endocytosis (LTD) in an activity-dependent manner [80]. We found that anesthesia exposure reduced the GluA3 expression levels in the membrane fraction. A previous study demonstrated that general anesthesia induced by intraperitoneal injections of pentobarbital sodium and chloral hydrate reduced membrane GluA3 and GluA1 in cortical neurons [81]. Our present study reproduced a similar phenotype in anesthesia-exposed WT mice and showed that reduction of membrane GluA3 had no effect on cerebellum-dependent motor learning. However, anesthesia-exposed App-KI mice showed impaired motor learning, but they did not show a reduction in membrane GluA3. As noted above, APP/PS1 mice and TgCRND8 mice showed a decline in LTD in cerebellar parallel fiber-Purkinje cell synapses, while anesthesia-exposed App-KI mice showed impairment in endocytosis with no reduction in membrane GluA3. These results indicate that dynamic changes in the number of GluA3 in the membranes of WT mice are required for adjustment to or protection against anesthesia. Meanwhile, App-KI mice may show deficits in this type of plasticity due to the accumulation of Aβ. Manipulative experiments using overexpression or knockdown of GluA3 in the cerebellum are needed to further elucidate these findings.

We recently reported, in a previous study, that desflurane induced no post-anesthetic effects in healthy young adult mice, except for mild short-term transient effects on motor coordination. Specifically, desflurane exposure one day before the behavioral tests increased the number of slips for only the first trial out of six trials in the balance beam test, whereas desflurane exposure three and seven days before the tests did not have any effects, suggesting that desflurane is safe for use in clinical settings with healthy young adults [35]. In the present study, motor coordination and motor learning function in aged WT mice were not affected by desflurane or sevoflurane. In contrast, both desflurane and sevoflurane exposure in App-KI mice caused impaired motor performance improvement in each trial. Anesthesia-induced motor learning deficits in App-KI mice indicate that anesthesia must be carefully used in patients with presymptomatic AD-like pathological alterations.

Elderly people with cognitive impairment, especially patients with AD, are potentially at a higher risk of falling than age-matched healthy controls [82, 83]. Deficits in higher-level cognitive functioning, such as executive dysfunction and anosognosia, are also risk factors for falling, in addition to age-related physical declines, such as sensory deficit and loss in muscle strength [84].　Furthermore, in a prospective cohort study, Stark et al. [85] demonstrated that individuals with presumptive preclinical AD without cognitive impairments had a higher probability of falling. These results are compatible with findings from other studies demonstrating motor impairment in animal models with pre-clinical AD [1, 58, 59, 65–67]. Further, anesthesia-induced prolonged deficits in motor learning in App-KI mice found in the current study suggest that the use of anesthesia in patients at-risk for and diagnosed with AD requires careful and subsequent follow-up.

There are two major limitations of this study that should be addressed in future research. First, although App-KI mice are currently one of the most practical animal models of AD, they do not fully replicate human AD pathology. They do not exhibit tauopathy, neuronal loss, or severe behavioral phenotypes [36]. In addition, this animal has been genetically modified for Aβ production, aggregation, and degradation, but Aβ clearance remains unclear. Therefore, further genetic manipulation is required. Second, in the current study, due to time, space, and life resource limitations, we were only able to assess three behavioral tests at the seven-day time point after anesthesia exposure. Previously, we systematically investigated general health, neurological reflexes, in addition to sensory, motor, attentional, emotional, sociability, and learning functions in healthy adult mice seven days after isoflurane [52] and desflurane [35] exposure using the same facilities and environment as the present study. Seven-day time point after anesthesia exposure was selected to allow for comparisons with our previous studies. For a more accurate behavioral phenotype description, it is necessary to evaluate using multiple timepoints and perspectives, including cognitive and other non-cognitive functions. Moreover, in the present study, we found that GluA3-containing AMPA receptors are involved in motor learning disabilities in anesthesia-exposed App-KI mice; however, the underlying molecular mechanisms of their involvement remain unclear.

In conclusion, desflurane and sevoflurane induced motor learning deficits in App-KI mice but not in WT controls. However, further studies are required to fully understand the neurophysiological basis of the effects of anesthesia on motor learning in AD animal models. Nevertheless, this is the first report to assess the effect of general anesthesia on motor learning in App-KI mice exposed to desflurane and sevoflurane.

## Supplementary Information

**Additional file 1: Fig. S1**. Effect of general anesthesia on crude fraction expression levels of AMPA receptors in the cerebellum in aged App-KI mice. Mean (+ SEM) protein expression ratio (normalized with the β-actin expression levels) of GluA1 (A), GluA2 (B), GluA3 (C) and GluA4 (D) subunits in the crude fractions in each group, WT-Air (n = 3), WT-Des (n = 3), WT-Sev (n = 3), App-Air (n = 5), App-Des (n = 5) and App-Sev (n = 5) group, are shown. Individual values were normalized with the mean values of WT-Air group as 1.

## Data Availability

The datasets supporting the conclusions of this article are included within the manuscript.

## Abbreviations

AD: Alzheimer’s disease
Aβ: Amyloid β peptide
SAD: Sporadic Alzheimer’s disease
FAD: Familial Alzheimer’s disease
App-KI: App^NL-G-F/NL-G-F^
WT: Wild type
PCR: Polymerase chain reaction
MAC: Minimum alveolar concentrations
ANOVA: Analysis of variance
LTD: Long-term depression
LTP: Long-term potentiation
