# Supplementary figures and images for: Assessments of prolonged effects of desflurane and sevoflurane on motor learning deficits in aged AppNL-G-F/NL-G-F mice
Source: Mol Brain. 2022 Apr 7;15:32. doi: 10.1186/s13041-022-00910-1 (PMC8988377; doi:10.1186/s13041-022-00910-1)

(A)

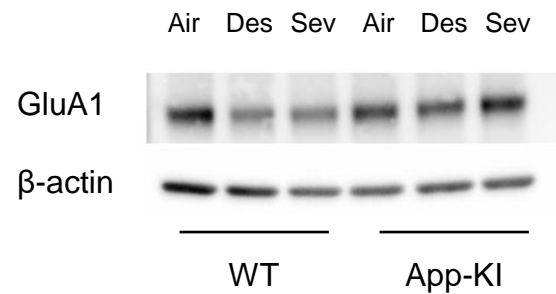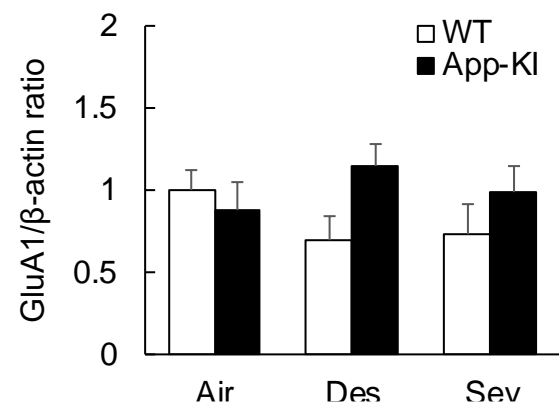

(B)

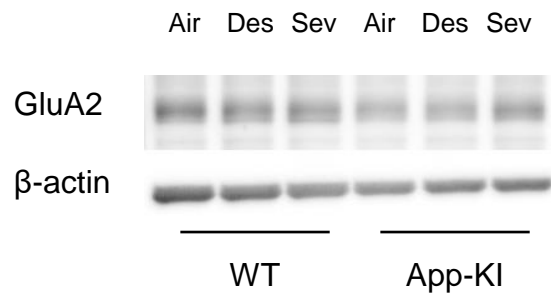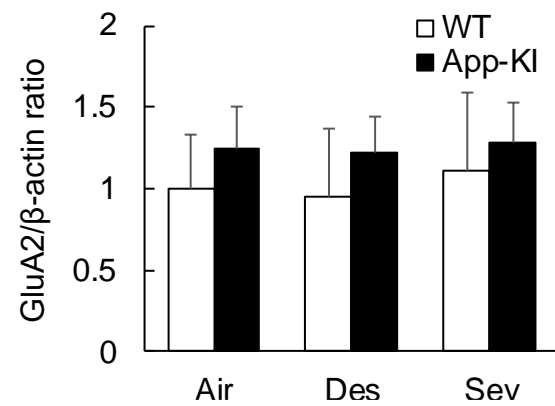

(C)

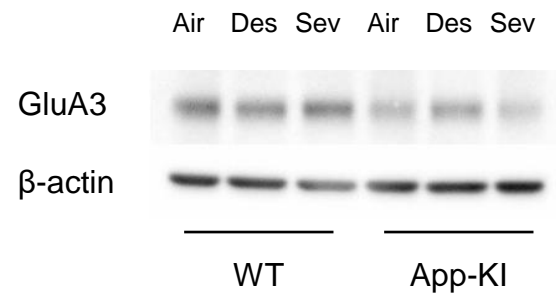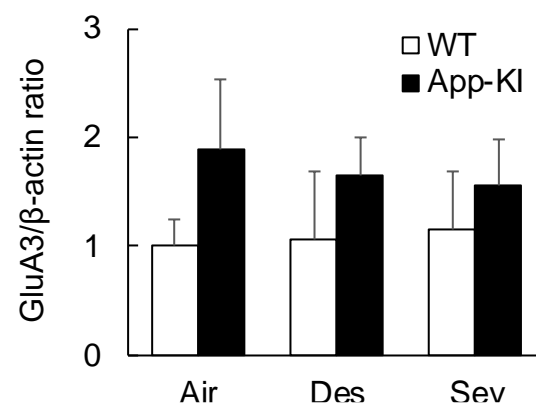

(D)

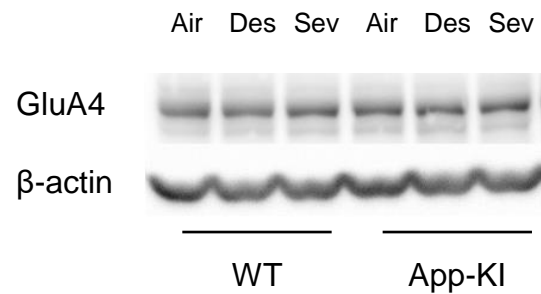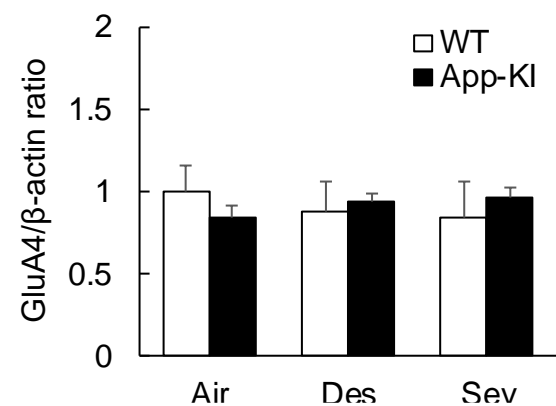

Supplement: Supplementary file 1 — Additional file 1: Fig. S1. Effect of general anesthesia on crude fraction expression levels of AMPA receptors in the cerebellum in aged App-KI mice. Mean (+ SEM) protein expression ratio (normalized with the β-actin expression levels) of GluA1 (A), GluA2 (B), GluA3 (C) and GluA4 (D) subunits in the crude fractions in each group, WT-Air (n = 3), WT-Des (n = 3), WT-Sev (n = 3), App-Air (n = 5), App-Des (n = 5) and App-Sev (n = 5) group, are shown. Individual values were normalized with the mean values of WT-Air group as 1. [file 13041_2022_910_MOESM1_ESM.pdf]
